# Chromosome-condensed G1 phase yeast cells are tolerant to desiccation stress

**DOI:** 10.15698/mic2022.02.770

**Published:** 2021-11-26

**Authors:** Zhaojie Zhang, Gracie R. Zhang

**Affiliations:** 1 Department of Zoology and Physiology, University of Wyoming, Laramie, WY, 82071, USA.; 2 Laramie High School, 1710 Boulder Dr. Laramie, WY 82070, USA.

**Keywords:** aging, cell cycle, chromosome condensation, desiccation tolerance, Saccharomyces cerevisiae

## Abstract

The budding yeast *Saccharomyces cerevisiae* is capable of surviving extreme water loss for a long time. However, less is known about the mechanism of its desiccation tolerance. In this study, we revealed that in an exponential culture, all desiccation tolerant yeast cells were in G1 phase and had condensed chromosomes. These cells share certain features of stationary G0 cells, such as low metabolic level. They were also replicatively young, compared to the desiccation sensitive G1 cells. A similar percentage of chromosome-condensed cells were observed in stationary phase but the condensation level was much higher than that of the log-phase cells. These chromosome-condensed stationary cells were also tolerant to desiccation. However, the majority of the desiccation tolerant cells in stationary phase do not have condensed chromosomes. We speculate that the log-phase cells with condensed chromosome might be a unique feature developed through evolution to survive unpredicted sudden changes of the environment.

## INTRODUCTION

As an anhydrobiote, the budding yeast *Saccharomyces cerevisiae* is capable of surviving extreme water loss [1–4]. Given the essential functions of water in biological systems, desiccation could impose multiple stresses, such as osmotic stress [5] and oxidative stress [6]. Cellular membrane and protein structures are also altered in response to loss of water [7, 8]. Some membrane proteins, such as the endoplasmic reticulum protein Ist2 plays an important role in preserving the molecular organization of the membrane [9]. A better understanding on how yeast cells mitigate desiccation stress is of great interest amid the global climate change. It may provide broad applications such as developing more drought tolerant crops. For example, yeast can be used for quick screening of plant desiccation tolerant genes due to its fast growth and easiness of genetic manipulation. Expression of *Arabidopsis thaliana* late embryogenesis abundant (LEA) proteins in yeast revealed that some, but not all of the LEA proteins enhanced desiccation tolerance in yeast [10]. Desiccated yeast cells could also potentially be used as a water-free biobank for long-term preservation of desiccation sensitive enzymes at room temperature [11].

Studies have shown that yeast, along with other anhydrobiotes, are rich in various desiccation stress effectors, such as non-reducing disaccharides, primarily trehalose [8], and hydrophilins, which are short, unstructured hydrophilic proteins [12]. These unique proteins and non-reducing sugars help stabilize and preserve both membrane and protein structure during the desiccation process [13, 14]. Heat shock proteins (HSPs) also play important roles in stress response, including response to desiccation. They act as chaperones for proper protein folding and prevent the aggregation and misfolding of proteins during stress. Hsp70, an ATP-dependent chaperone essential for protein folding, was upregulated in the desiccation tolerant *Klebsormidium* strain under desiccation stress [15]. Knockdown of Hsp70 gene reduces the viability of desiccated cysts of *Artemia* [16]. A genome wide screening in budding yeast revealed that respiration is a prerequisite in acquiring desiccation tolerance [17], and it is likely associated with the dynamic changes of mitochondria [18]. In yeast, due to starvation stress or lack of nutrient, stationary cells become more resistant to different stresses, via dramatic decrease in overall growth, enriched lipids, trehalose and proteins, and thickened cell wall that are necessary to encounter heat, cold, or desiccation stress [8, 19]. Our recent work showed that membrane and lipid metabolism also play an important role in desiccation resistance in yeast [20]. We showed that desiccation causes ER stress and unfolded protein response, which triggers an increased membrane and lipid metabolism. It, in turn, may provide cells with energy and possibly metabolic water that are essential for enzymatic activities in desiccated cells [21].

Under optimal growth condition, yeast cells grow relatively fast and can progress through a cell cycle in about 90 minutes. The specific stage of a cell in a cell cycle may also play a role in desiccation tolerance [15, 22]. During the cell cycle, multiple checkpoints are put in place to ensure the fidelity between successive generations and prevent the formation of genetically defective cells. Failure of any checkpoint may result in uncontrolled cell proliferation, or cell death [23–25]. G1 checkpoint is the main decision point, where it determines whether a cell to divide or not. Once it passes the G1 checkpoint and enters S phase, the cell is committed to an irreversible division [26]. If the environment becomes non-permissible (such as heat, cold or drought), or the cell encounters irreparable damages (such as DNA damages) [27], the dividing cell may undergo regulated cell death [28]. As a result, dividing cells are more vulnerable to both internal and external (environmental) stresses, compared to resting cells. In yeast, while stationary cells are highly tolerant to desiccation stress, exponentially growing cells are very sensitive to desiccation [17].

Cell cycle arrest and reduction of cell division caused by desiccation have been reported in both desiccation tolerant plants [29, 30] and algae [15, 31]. Upon dehydration, DNA replication is repressed and new cycle of cell division is arrested. As a result, all cells are in G1 phase [15]. Transcriptomic analysis of the desiccation-tolerant microalgae *Klebsormidium* [15] revealed a down-regulation of many cell cycle associated transcripts, including transcripts for spindle assembly checkpoint proteins and the condensin complex, which is required for establishment and maintenance of chromosome condensation and chromosome segregation [32]. In addition, other transcription and translation related transcripts are down-regulated [15].

In addition to the lack of abundant trehalose [21], log-phase cells contain more dividing cells, which could contribute to its low survival under desiccation. In this study, we investigated cell cycle in relation to desiccation tolerance in the yeast *S. cerevisiae*. We found that in exponentially growing cells, only a small portion of G1 phase and replicatively young cells were tolerant, while cells in all other phases of the cell cycle were sensitive to desiccation stress. The desiccation tolerant G1 cells share certain features of G0 cells, such as chromosome condensation and low metabolic level. Our study suggests that yeast may have revolutionarily evolved a survival mechanism in response to unpredicted harsh environmental conditions during their normal cell cycle and normal growth.

## RESULTS AND DISCUSSION

### Dividing cells are sensitive to desiccation stress

Studies have shown that the desiccation tolerance of yeast from a stationary-phase culture is much higher than that from an exponential culture, which ranges from one in a million [17] to about 5–10% [20, 33]. Faster desiccation usually results in lower survival rate while slower desiccation provides a higher rate of desiccation tolerance [34]. One possible reason is that faster desiccation may cause more membrane damages, as suggested by a significant increase of acid phosphatase during fast drying [34, 35]. In plants, rapid desiccation causes microtubule depolymerization [29]. Other factors, such as the solution/buffer and the volume of cell suspension prior to desiccation, may also significantly affect the survival rate [8, 20, 21]. Slower desiccation, on the other hand, may provide a permissive condition to many enzymes that could be harmful to cells. The desiccation tolerant cells may have a mechanism to mitigate these enzyme activities, such as silencing gene expression. Scavenging mechanisms such as synthesis of antioxidants are also elevated in desiccation tolerant organisms in response to the increase of harmful reactive oxygen species (ROS) during dehydration [30, 36].

**Figure 1:**
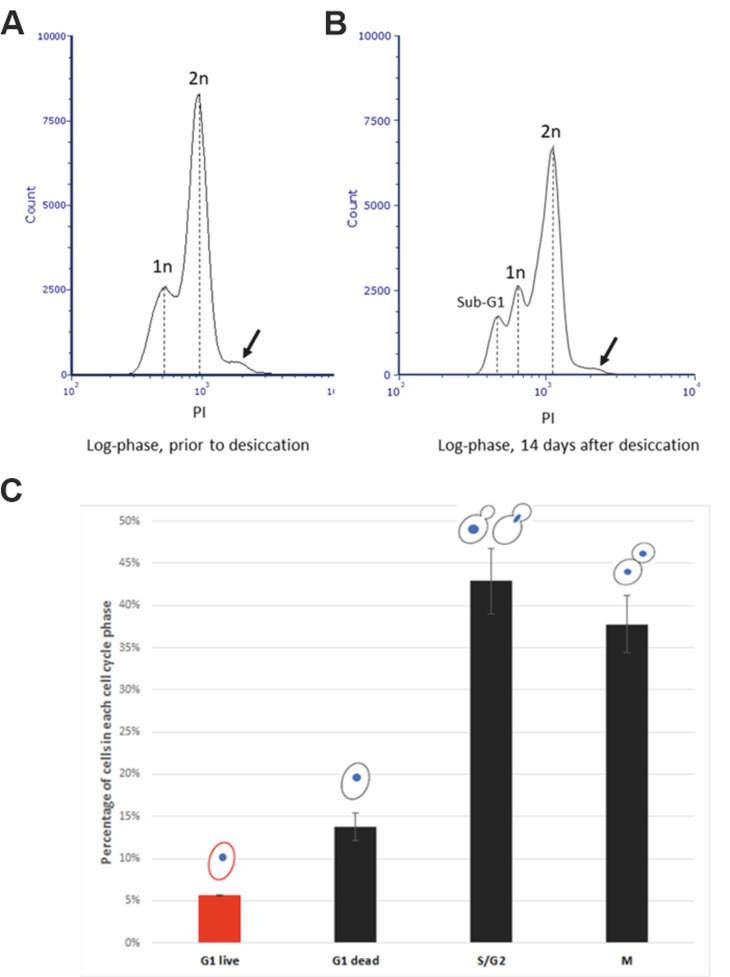
FIGURE 1: **(A, B)** Flow cytometry analysis of exponential cells (overnight culture) prior to **(A)**, and 14 days after **(B)** desiccation. Cells were fixed with 70% ice cold ethanol, stained with propidium iodide (PI) Cell Cycle solution, and analyzed using an Bio-Rad S3e cell sorter. Arrow indicates the small population of cells that had higher DNA content. 1n and 2n indicate one copy and two copies of DNA, respectively. **(C)** Percentage of live/dead cells in different cell cycle phases of exponential cells after 14 days of desiccation. Live and dead cells were identified by PI staining. Cell cycle status was determined by images of bright field and fluorescence brightener-28 stained cells. Approximately 400 cells were counted from each experiment and the data were presented as mean ± standard error from three independent experiments.

**Figure 2:**
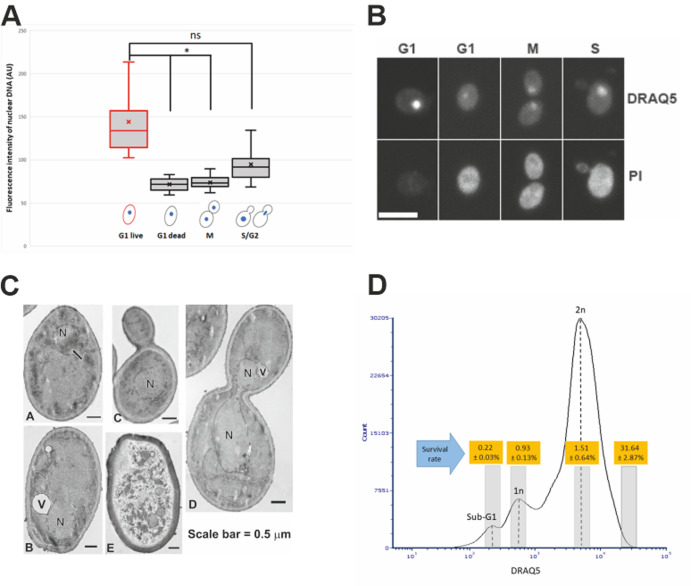
FIGURE 2: Chromosome condensation in desiccated G1 log-phase cells. **(A)** Measurement of DNA content of live/dead log-phase cells (after 14 days of desiccation) in different cell cycle phases. Approximately 400 cells were counted from each experiment and the data presented here are the sum of three independent experiments. *: p < 0.05; ns: not significant. **(B)** Representative confocal microscopy images of log-phase cells at different cell cycle stages after 14 days of desiccation, showing higher fluorescence intensity (indicated by DRAQ5 staining) in live G1 phase cells (indicated by negative PI staining). The dead G1, S and M phase cells (indicated by positive PI staining) had lower DNA fluorescence intensity (indicated by DRAQ5 staining). Scale bar = 5 μm. **(C)** Transmission electron microscopy images of log-phase cells after 14 days of desiccation (A); a G1 phase cell with condensed chromosome (arrow) (B); a G1 phase cell with no chromosome condensation (C); a S-phase cell with no chromosome condensation (D); a G2 phase cell with no chromosome condensation (E). a dead cell. N = nucleus, V = vacuole; **(D)** Cell sorting and cell viability of sorted log-phase cells after 14 days of desiccation. The histogram shows the flow cytometry analysis of the DRAQ5 stained log-phase cells. The grey bars on the histogram indicate cells that were sorted and tested for viability. Viability was tested by plating assay. Data were presented as mean ± standard error from three independent experiments.

We speculate that the cell cycle status could also be a factor that causes the low desiccation tolerance of the exponentially growing (log-phase) cells. To test this, we first examined the percentage of cells in different stages of the cell cycle of an exponential culture. Flow cytometry analysis revealed that about 25% of cells were in G1 phase, while the remaining were in either S, G2 or M phase prior to desiccation (**Fig. 1A**). After 14 days of desiccation, a similar distribution of the cell cycle stages was observed, except that about 10% of cells were in Sub-G1 stage (**Fig. 1B**), suggesting these cells were in the stage of regulated cell death (RCD) [28]. While flow cytometry is capable of analyzing large number of cells, it cannot identify individual cells and their cell cycle status. We used laser scanning confocal microscopy to examine individual live/dead cells and correlate with their cell cycle status. Propidium iodide (PI) staining showed a 6% survival rate, similar to our previous report [20]. We found that all live cells were in G1 phase. No live cells were observed in S, G2 or M phase (**Fig. 1C**). It is worth noting the seemly discrepancy between the percentage of RCD cells revealed by flow cytometry (**Fig 1B**) and the dead cells detected by confocal microscopy (**Fig. 1C**). For cytometry analysis, cells were fixed with ethanol and PI stained the nucleus. It allows the identification of the sub-G1 cells (RCD cells). For confocal microscopy imaging, cells were not fixed and PI stains primarily the cytoplasm of dead cells, while live cells were not stained (**Fig. 2B**).

### Desiccation tolerance correlates with chromosome condensation

Among the G1 cells, about 30% were survived from the 14-day desiccation (**Fig. 1C**). To examine the possible differences between the surviving and dead G1 cells, we used DRAQ5, a fluorescent DNA dye that stains both live and dead cells, to stain the nucleus. We found that the nuclei of the desiccation tolerant G1 cell were much brighter than that of the dead G1 cells. We further quantified the nuclear DNA using ImagJ software. The DNA content of single nucleus from M phase was defined as 1N. We found that the DNA content of S/G2 phase cells ranged from 1N to 2N. The dead G1 cells had a similar DNA content compared to the M phase single nuclear DNA (1N). However, all the live G1 cells had a much higher DNA fluorescence intensity, equivalent to 1.5N to 2.2N (**Fig. 2A**, **2B**), which is significantly higher than the M phase or G1 dead cells (p < 0.05). Flow cytometry analysis also suggests a possible small population of cells that had higher DNA fluorescence intensity (**Fig. 1A**, **1B**, arrow). Considering G1 cells should have only 1N DNA content, these results suggest that chromosomes of the desiccation tolerant cells were condensed, similar to G0 cells found in stationary phase cells [37, 38].

An early study using transmission electron microscopy (TEM) has shown yeast chromosome condensation during early stage of desiccation [39], and it is believed that this is a protection mechanism for preserving the nuclear DNA during desiccation [2, 3, 34]. We used TEM to further examine the nuclear structure of log-phase cells after 14-day desiccation. Chromosome condensation was observed in 3.67% of G1 cells, while no chromosome condensation was observed in dividing cells. Dead cells were also observed after desiccation (**Fig. 2C**).

To check whether this chromosome condensation is induced during the desiccation process, we examined the DNA content of log-phase cells (overnight culture) prior to desiccation. A similar percentage of G1 cells was found to have higher DNA fluorescence intensity, which was about 1.3 times higher than the average mean value of M phase DNA. However, the level of condensation is significantly less than in desiccated live G1 cells (p < 0.05). To ensure this observation is from true log phase cells, we further examined cells from a seven hr culture. G1 cells with higher DNA fluorescence intensity were also observed, similar to the overnight culture. These results suggest the presence of G0-like cells in exponential culture before the desiccation stress, and the chromosomes of these G0-like cells were further condensed during the desiccation process, making them more resistant to desiccation stress. This may explain in part, that a slower desiccation process significantly improves the survival rate [17, 20], while in a fast desiccation process, these G0-like cells do not have an opportunity/time to further condense their chromosomes, making them susceptible to desiccation.

To further confirm that chromosome condensation positively correlates with desiccation tolerance, a cell sorter was used to separate and collect different populations of the desiccated log-phase cells from different cell cycle stages. We showed that after 14 days of desiccation, cells with higher DNA content reached a survival rate of almost 32%, while the 1N and 2N cells had a survival rate of 1.5% or less (**Fig. 2D**). This further confirmed that cells with higher DNA content (indicated by higher fluorescence intensity) were more resistant to desiccation. Proper gating was applied to ensure cells with higher DNA content were not doublets (see Materials and Methods for details). One limitation of the cell sorting is that the DRAQ5 staining (without fixation) could not identify and separate cells only in G1 phase with higher DNA content. As a result, the sorted cells with higher DNA content likely included cells from both G1 (live) and S/G2 (dead) stage, resulting in a lower (31.64%) survival rate. We anticipate the survival rate would be much higher, if the sorted cells contained only G1 cells with higher DNA fluorescence intensity.

Cell sorting may provide other potential applications in yeast research. For example, to sort the sub-G1/G0 cells, which is a better-defined sub-population. Cell sorting may also be used to synchronize cell culture by separating the G1, or M phase cells. It is simple and can simultaneously separate cells from multiple phases, and introduces less damages caused by chemical inhibitors [40].

Desiccation tolerance is affected by many factors, such as the physiological state and metabolic composition of the cell. Studies have shown the metabolism of carbohydrate is dramatically altered in response to desiccation. Starch degradation is enhanced and sucrose concentration increased upon desiccation [41, 42]. The increased sucrose may function as an osmoprotectant to retain water within the cell and protect both proteins and membrane structure [43]. Changes of lipid metabolism have also been reported in plants [44] as well as in yeast [20]. Yeast cells gain their tolerance via accumulation of desiccation-related substances including unstructured hydrophilic proteins [12] and non-reducing disaccharides [8], which help stabilize the membrane and protein structure during desiccation. Accumulation of these substances normally requires yeast cells growing into stationary phase, when less nutrients become available. It is intriguing how a small portion of the log-phase G1 cells obtain their desiccation resistance. While the condensed chromosomes could help preserve the nuclear DNA [2, 3, 34], it remains unknown how membrane and proteins are being stabilized during desiccation in the desiccation tolerant log-phase G1 cells.

### Chromosome-condensed cells share certain features of G0 cells

**Figure 3:**
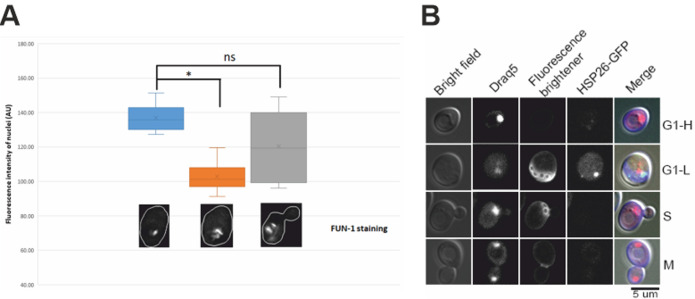
FIGURE 3: **(A)** Quantified DNA fluorescence intensity by DRAQ5 staining (top bar graph) in relation to metabolic level (FUN-1 staining, bottom) in log-phase cells without desiccation. It shows that G1 cells with higher DNA fluorescence intensity (by DRAQ5 staining) had a lower metabolic level (single crystal by FUN-1 staining) (left), while G1 cells with lower DNA fluorescence intensity had more than one FUN-1 crystals (middle), and S phase cells also had multiple FUN-1 crystals (right). Approximately 400 cells were counted from each experiment and the data presented here are the sum of three independent experiments. *: p < 0.05; ns: not significant. **(B)** HSP26-GFP localization in log-phase cells without desiccation. Hsp26-GFP was observed in G1 cells with low DNA content (G1-L), but not in G1 cells with higher DNA content (G1-H), S-phase (S) or M-phase cells (M).

One unique feature of G0 cells is their relatively low metabolic activity. We used the fluorescent dye FUN-1 to check the metabolic level of the log-phase cells prior to desiccation. FUN-1 forms red cylindrical intravacuolar structures (CIVS) in live and metabolically active cells [45]. The number and size of the CIVS proportionally correspond to cell’s metabolic level. Our results showed that G1 cells with higher DNA fluorescence intensity had relatively small number of CIVS compared with G1 cells with 1N DNA, or S phase cells (**Fig. 3A**), suggesting that these G1 cells with condensed chromosomes possess features of stationary G0 cells.

Another feature of the quiescent G0 cells is the expression of a subset of genes, such as *HSP26* [46]. We examined the *HSP26*-GFP expression in log-phase cells prior to desiccation. GFP Foci were observed mostly in G1 cells that had low DNA contents, especially in replicatively old G1 cells. Little GFP foci were observed in G1 cells with high DNA contents, or S-/M-phase cells (**Fig. 3B**), suggesting that desiccation tolerant G1 cells do not share all characters of quiescent G0 cells.

### Stationary G0 cells may possess different mechanism for desiccation tolerance

**Figure 4:**
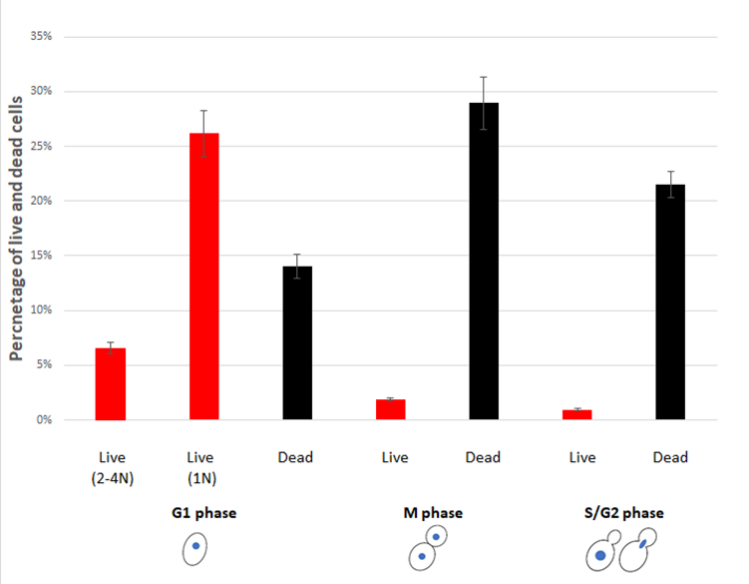
FIGURE 4: Percentage of live and dead stationary cells in different cell cycle phases after 14 days of desiccation. Two different population of live G1 cells were observed, one with high DNA fluorescence intensity (equivalent to 2-4N), and the other with 1N DNA fluorescence intensity. Live and dead cells were identified by PI staining. Cell cycle status was determined by images of bright field and fluorescence brightener-28 stained cells. Approximately 400 cells were counted from each experiment and the data were presented as mean ± standard error from three independent experiments.

G0 cells are normally obtained by growing yeast cells in liquid medium to stationary phase (three or more days in rich media). To further explore the possible connection between condensed chromosomes and desiccation tolerance, we examined the desiccation tolerance and DNA content of stationary cells (growing in liquid YPD for three days). Consistent with our previous finding, the three-day stationary cells were more resistant to desiccation than log-phase cells [20]. Similar to log-phase cells, a majority of the surviving cells in the three-day culture were in G1 stage. However, not all surviving cells had higher DNA fluorescence intensity. Surprisingly, the percentage of cells that had higher DNA fluorescence intensity was similar to that of log-phase cells; the remaining surviving cells had a DNA content similar to M-phase cells (∼1N). Regarding condensed chromosomes, the condensation level was much higher than that of the log-phase cells, equivalent to 2N to 4N of M-phase cells (**Fig. 4**), suggesting that chromosomal condensation is promoted upon entering the stationary phase. These results also suggest that stationary cells possess two different mechanisms to counter desiccation stress, one is “inherited” from the exponential cells with condensed chromosomes; the second is acquired via starvation due to the lack of nutrient. While a majority of cells acquire their desiccation tolerance through starvation, during which cells may accumulate trehalose and desiccation-related proteins, the desiccation tolerance induced by chromosome condensation may play a critical role in evolution to preserve the species from extinction during unexpected environmental stress under normal growth conditions. It would be interesting to see if this intrinsic feature for desiccation tolerance is adapted to other stresses, such as oxidative stress.

### Replicative lifespan plays a role in desiccation tolerance

**Figure 5:**
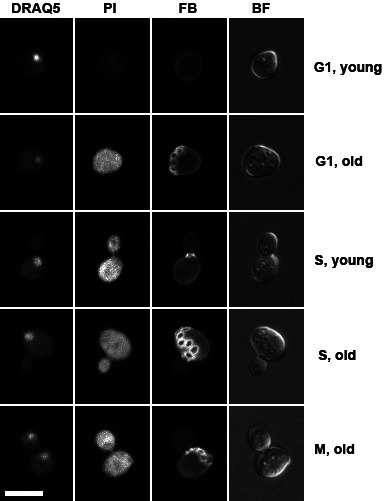
FIGURE 5: Confocal microscopy images of fluorescence stained log-phase cells after 14 days of desiccation, showing replicatively aged cells were more sensitive to desiccation. DRAQ5 stains nucleus, propidium iodide (PI) stains dead cells, fluorescence brightener 28 (FB) stains cell wall and bud scars, bright field (BF) shows the overall structure of the cell. Scale bar = 5 μm.

The lifespan of the budding yeast is measured in two ways: chronological lifespan (CLS) and replicative lifespan (RLS) [47]. CLS measures how long a cell can stay alive, while RLS refers to how many times a cell can divide or produce offspring. For log-phase cells, CLS is limited to overnight growth. The RLS is less homogenous. Using a fluorescence dye that stains cell wall and bud scars, we observed cells with from zero to multiple bud scars in an overnight culture. We then compared the number of bud scars between live and dead G1 cells. We found that all live cells had either no or one bud scar, while dead G1 cells had zero to up to five bud scars (**Fig. 5**). This result suggests that the desiccation tolerant cells were all derived from replicatively young cells.

For stationary cells, the intrinsically desiccation-tolerant cells (cells with higher DNA fluorescent intensity) were all found to have no or one bud scar, similar to log-phase cells. Over 90% of the cells with acquired desiccation tolerance (lower measured DNA content) were also found with no or one bud scar. Only less than 10% of these cells had two bud scars, suggesting that the acquired desiccation tolerance occurs also primarily in replicatively young cells.

Yeast cells undergo asymmetric cell division, in which the mother cell ages, while the newborn daughter cell is rejuvenated [47]. As a result, replicatively aged cells are more prone to environmental stress [48], and they ultimately die in an apoptotic fashion [49]. Consistent with these reports, we demonstrated that the replicative young cells in both exponential and stationary cultures are more desiccation tolerant.

In summary, our study suggests that even under favorable growing conditions, a small number of replicatively young cells are prepared to encounter environmental stresses. Their gene expression and metabolic level are likely low due to condensed chromosomes. This undoubtedly gives yeast an advantage in evolution to survive in an unpredicted harsh environment while growing under normal conditions.

## MATERIALS AND METHODS

### Yeast strain and desiccation conditions

The yeast *Saccharomyces cerevisiae* wild type strain BY4742 (Matα *his3*Δ *leu2*Δ *met15*Δ *ura3*Δ) and its derivative HSP26-GFP were purchased from ThermoFisher (Waltham, MA). Cells were grown in 15 ml round-bottom glass tubes (Fisher Scientific, Waltham, MA) to log- (14 hrs) or stationary phase (three days) in YPD medium (1% yeast extract, 2% peptone and 2% dextrose) at 30°C with constant shaking at 250 rpm. Cells were centrifuged (× 1,000 g for five min) and the culture medium was discarded. The culture tubes without caps were placed in a humid chamber (23°C, 50% relative humility) and cells were allowed to desiccate for 14 days [20].

### Flow cytometry analysis

For flow cytometry analysis, desiccated cells were resuspended in phosphate buffered saline (PBS, pH 7.4) at room temperature, vortexed for 30 sec, then diluted to a final concentration of 5 × 10^6^ cell/ml. Cells were fixed immediately with 70% ice cold ethanol for 15 min, then washed once with PBS. The cells were then stained with 500 μl of PI Cell Cycle solution (CSK-0112, Nexcelom, Lawrence, MA) for 40 min in a 37°C incubator. Cells were resuspended in PBS and analyzed using an S3e cell sorter (Bio-Rad, Hercules, CA). For control, log-phase cells without desiccation were fixed and analyzed in the same way as the desiccated cells. The propidium iodide (PI) staining was excited by a 561 nm laser with an emission filter of 615/25 nm. To exclude cell aggregates, the resuspended cells were filtered through a 20-μm sterile nylon filter. Cell debris was excluded by plotting forward scatter (FSC) area vs side scatter (SSC) area. Particles that were too small (debris) were removed by gating. Cell doublets were discriminated by plotting forward scatter (FSC) height vs FCS area. Doublets have increased area whilst similar height to single cells, and doublets were removed by gating. Both FSC and SSC were plotted on linear scales, while fluorescence measurement was plotted on log scale due to intensity differences were too significant to be placed on a linear scale. The cytometry data were analyzed using the FCS express 7 software (De Novo Software, Pasadena, CA).

### Laser scanning confocal microscopy imaging

Desiccated cells were resuspended in PBS as above. Cells were then stained with the following fluorescence dyes for three min in dark; PI (final concentration = 2 μg/ml, ThermoFisher Sci., Waltham, MA), DRAQ5 (final concentration = 5 μM, ThermoFisher), and Fluorescence Brighter (FB)-28 (final concentration = 0.1 mg/ml, Sigma-Aldrich, St. Louis, MO). For microscopic imaging, a 13 mm diameter, 0.12 mm deep imaging spacer (ThermoFisher) was placed on a microscopic slide. 80 μl of 1.5% low-melting agarose (pre-heated to 40°C) was placed in the spacer well, which was then covered with a coverslip. The microscopic slide chamber was placed at 4°C for 30 min, to solidify the agarose. The coverslip was then removed, and 10 μl of stained cells were pipetted onto the agarose, then coverslipped. The stained cells were imaged using a Zeiss 980 laser scanning confocal microscope (Zeiss, NY, NY). Z-stacks were taken at a 0.3 μm step size and z-projections were used for quantifying DNA content using ImageJ software (https://imagej.nih.gov/ij).

For FUN-1 staining, 20 μl of 1 mM FUN-1 stock solution (final concentration=20 μM) (ThermoFisher) was added to 1 ml of cells. Cells were incubated at 30°C in dark for 30 min. Cells were then imaged as described above.

### Cell cycle determination

The status of cell cycle was determined manually by confocal microscopy images. Images of bright field and fluorescence brightener-28 stained cells were used to determine the budding and DRAQ5 stained cells to determine the nuclei. The cell cycle status was determined as: G1 phase: unbudded cells; S phase: small-budded cells with bud < 50% of the mother cell; G2 phase: large-budded cells with single nucleus; for practical reasons, S phase and G2 phase were grouped together; M phase: large-budded cells with two separate nuclei. Approximately 400 cells were counted from each experiment and the experiment was repeated 3 times.

### Transmission electron microscopy (TEM) observation

TEM Samples were prepared as previously described [20]. Thin sections of 60 nm were cut with an MTXL ultramicrotome (RMC Boeckeler, Tucson, AZ) using a diamond knife. Sections were observed with a Hitachi H-7650 TEM (Hitachi High-Tech America, Schaumburg, IL).

### Cell sorting

Cells were sorted using a Bio-Rad S3e cell sorter. Desiccated cells were rehydrated in PBS at room temperature, vortexed for 30 sec, and stained with 5 μM DRAQ5 for three min. No fixation was performed. Cells were analyzed using a 488 nm laser for excitation and 655 nm long-pass filter for emission. Cells at different cell cycle were selected based on the histogram and sorted into different tubes, using the “Single Cell” mode to ensure only single cells were collected. Sorted cells were plated on YPD plates (1,000 cells/plate) and cultured for three days at 30°C for two to three days and the number of colonies were counted. G1 phase cells of non-desiccated log-phase cells were sorted and used as a control (500 cells/plate). Three plates were used for each sorted cells and the experiment was repeated three times. The survival rate was calculated as:

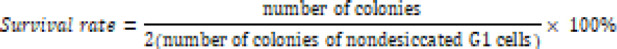


### Statistical analysis

Quantification was performed from three independent experiments. Data were expressed as mean ± standard error (SD). T-test was used to compare the statistical difference tween groups. p < 0.05 was considered statistically significant.

## Abbreviations:

CIVS: – cylindrical intravacuolar structures;
CLS: – chronological lifespan;
HSP: – heat shock protein;
LEA: – late embryogenesis abundant;
RCD: – regulated cell death;
RLS: – replicative lifespan.
